# Cystic fibrosis-related diabetes in the era of modern treatment using CFTR modulators in pediatric patients—a systematic review

**DOI:** 10.3389/fped.2025.1688862

**Published:** 2025-11-17

**Authors:** Anna Pietrzykowska, Jerzy Darmoń, Katarzyna Szymocha, Wiktoria Donocik, Mateusz Tarasiewicz, Julia Stadnik, Przemysława Jarosz-Chobot

**Affiliations:** 1 Student Scientific Association at the Department of Children’s Diabetology and Lifestyle Medicine, Medical University of Silesia, Katowice, Poland; 2 Department of Children’s Diabetology and Lifestyle Medicine, Medical University of Silesia, Katowice, Poland

**Keywords:** cystic fibrosis, CFTR modulators, CFRD, elexacaftor, ivacaftor, tezacaftor, lumacaftor, pediatric patient

## Abstract

**Background:**

Cystic fibrosis-related diabetes (CFRD) is a common comorbidity in cystic fibrosis (CF), particularly in the pediatric population. As cystic fibrosis transmembrane conductance regulator modulators (CFTRm) become widely used, there is growing interest in their potential metabolic benefits. This systematic review evaluates the effects of CFTR modulators on glucose metabolism and glycemic control in children and adolescents with CFRD.

**Materials and methods:**

Following PRISMA guidelines, we conducted a literature search in PubMed, Scopus, and Medline for all papers until 2025. Eligible studies included clinical trials, observational studies, chart reviews, and case reports focusing on pediatric patients receiving CFTRm.

**Results:**

From almost 653 initially identified records 5 studies met inclusion criteria - 1 clinical trial, 2 observational studies and 2 case reports. Evidence suggests CFTRm may improve glucose tolerance and insulin secretion in some pediatric patients, particularly in those with preserved β-cell function or early-stage CFRD. However, results varied across studies with some showing no significant improvements in glycemic control.

**Conclusions:**

While early findings suggest CFTR modulators may offer metabolic benefits and potentially delay or reduce the need for insulin therapy in children CFRD, current evidence is limited. Larger, pediatric-focused clinical trials with standardized glycemic outcomes are essential to determine the long-term efficacy and safety of CFTRm in managing or preventing CFRD.

## Introduction

While CFRD shares some characteristics with both type 1 and type 2 diabetes, it is classified as a distinct type of diabetes. CFRD affects about 9% of children aged 10–15 years, worsening lung function and life expectancy (1). The most common mutation in CF is F508del, which occurs in about 65% of individuals with CFRD (2, 3). Characterizing mutations is essential for accurate diagnosis and effective treatment.

Recent medical advances have led to the development of therapies that alleviate symptoms, extend life expectancy, and improve quality of life in patients with cystic fibrosis (4). Current treatment of CFRD typically includes nutritional support, insulin therapy, and non-insulin pharmacological approaches (5, 6). While conventional strategies aim to manage the downstream consequences of CFTR dysfunction, highly effective CFTR modulator therapy (HEMT) offers a promising approach by targeting the molecular defect itself (6). As median survival in cystic fibrosis continues to rise, an increasing number of children with CF will reach adulthood, when the risk of developing CFRD is significantly higher. Nevertheless, the median age at diagnosis is around 3 months, and children currently make up nearly half of the CF population (2, 3, 7). Consequently, the need for treatments, including those for CFRD, is increasing, so initiating effective therapies early in life, particularly CFTR modulators, which target the root cause of CF. Early intervention may reduce the future burden of CFRD and improve long-term outcomes in this growing pediatric population. It emphasizes the importance of effective and early treatments that address the underlying cause of CF in children, particularly CFTRm. Administering these treatments early in childhood may further reduce mortality rates in adults who already have a better prognosis for this disease.

This systematic review aims to collect, analyze, and summarize research papers related to patients with CFRD, patients diagnosed with type 1 diabetes (T1D) who also have CF, patients with CF who do not exhibit recognized glycemic disorders or are at risk of developing CFRD in the era of modern targeted treatment with the use of CFTRm and its influence on metabolic and glycemic control compared to insulin therapy. Specifically, we seek to evaluate the effects of this therapy in children and adolescents from birth to 18 years old.

## Materials and methods

This review was conducted following the PRISMA statement for systematic reviews (2). The literature search began on December 18, 2024, and lasted until March 29, 2025. It was applied to three electronic databases: PubMed (all papers until 2025), Scopus (all papers until 20–25), and Medline (all papers until 2025). We focused exclusively on the final publication stage of papers for this review. Our inclusion criteria encompassed randomized controlled trials, controlled clinical trials, observational studies, case reports, and chart reviews of the patients. We opted to include case reports because they provide valuable insights and are the first in this topic to show precisely the impact of treatment with CFTRm in all children and adolescent groups. These reports have the potential to inspire new research and enhance clinical practices for treating children and adolescents with CFRD. Furthermore, they are potentially relevant for patients with CF who do not exhibit recognized glycemic disorders or who are at risk of developing CFRD while being treated with CFTRm. We excluded reviews, letters, commentaries, editorials, book chapters, short surveys, notes, conference papers, and guidelines from our search. Only papers written in English were included in this review. The following keywords were employed: “cystic fibrosis transmembrane receptor modulators”, “cystic fibrosis related diabetes”; elexacaftor; ivacaftor; tezacaftor; lumacaftor; child*; youth*; adolescent*; teen*; infant*; neonate*; newborn*. Additionally, we utilized extra search terms in the PubMed and MEDLINE databases, including “full text”, “free full text”. For the Scopus database, the additional search terms included the “subject area - Medicine” and “all open access”.

**Figure 1:**
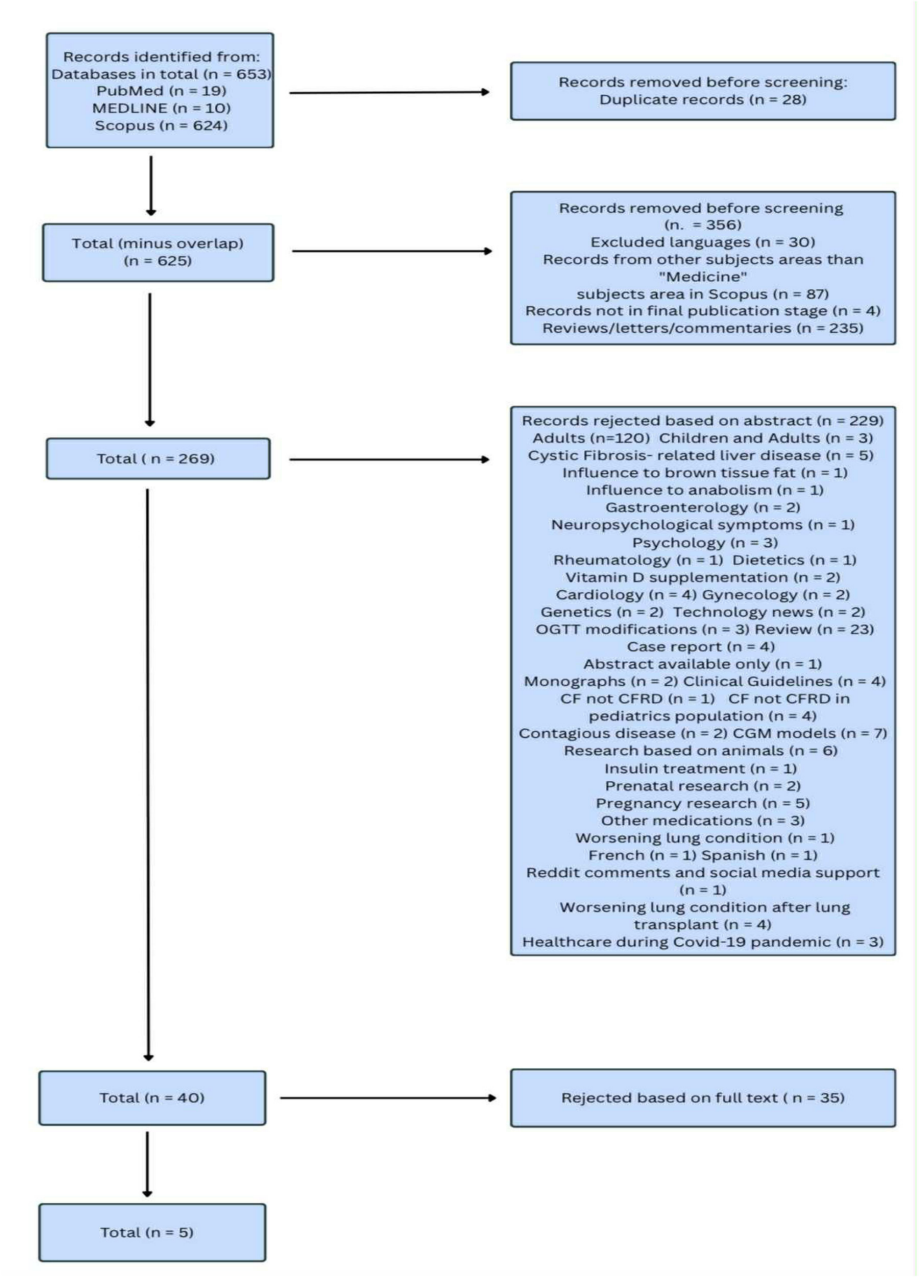
Methodology of the systematic review.

The reviewers performed an eligibility evaluation independently on the three different online databases. The study obtained a total of 653 articles - 19 articles from PubMed and 10 from the MEDLINE database, as well as 624 papers in the Scopus online database. All the records were screened to find eventual duplicates, and reviewers excluded 28 papers. 356 records were removed before screening based on the chosen criteria. The 269 remaining ones were screened by title and abstract, and 229 of them were excluded. Disagreements among the reviewers were resolved through consensus. After the selection process, 5 manuscripts in total were chosen for this review, including 1 clinical trial, 2 case reports, and 2 retrospective observational studies (Table 1; Figure 1).

**Table 1:** Summary table of studies meets inclusion criteria.

Author, year of publication	Study type	Sample (age group)	CFTR modulator	Main metabolic findings	Direction of effect	Comments
Bellin et al., 2013 (8)	Clinical trial (pilot study)	*n* = 5 (6–52 years); 1 pediatric CFRD	Ivacaftor	Prolonged-phase insulin secretion nearly doubled (1,485->2,955 mU/L min); OGTT glucose unchanged	↑insulin secretion (no change in HbA1c)	Suggest that preserved B-cell mass may respond to CFTR modulator correction in early phase of CFRD
Li et al., 2019 (9)	Observational study	*n* = 9 (median age 12.7 years); 1 with CFRD	LUMA/IVA^a^	HbA1c and fasting glucose increase after therapy; no glycemic improvement	↓glucose control	Suggest LUMA/IVA not metabolically beneficial
Korten et al., 2022 (10)	Observational study (pilot study)	*n* = 16 (median age 13.8 years); 2 with CFRD	ETI^a^	OGTT glucose decrease at 60, 90, 120 min (*p* < 0.05), insulin & C-peptide decrease; HbA1c unchanged	↑glucose tolerance	No HbA1c change; short term follow-up (4–6 weeks)
Park et al., 2024 (1)	Case report	*n* = 7 (>12 years); 7 with CFRD	ETI	4/7 discontinued insulin, 2 reduced doses; 1 needed increase; glycemia stable or improved	↑endogenous glucose control, ↓exogenous insulin need	Some developed hypoglycemia post-insulin withdrawal
Stekolchik et al., 2022 (11)	Case report	*n* = 1 (16 years old, female)	ETI	HbA1c rise from 7.8% to 8.7% after 10 months, weight gain observed	↓glucose control	Possible nutritional factors; unclear causality

a LUMA/IVA, lumacaftor-ivacaftor; ETI, elexacaftor-tezacaftor-ivacaftor.

## Results

The information needed was gathered from each included manuscript. We have taken into consideration the characteristics of participants, such as age, sex and diabetes duration, as well as treatment with CFTRm (type of drug used, treatment duration and study protocol), baseline metabolic status of participants and glycemic control parameters after therapy initiation. The data were analyzed and compiled through a narrative analysis. Findings from the research were organized based on the type of paper. When data were collected from a group that was not divided into age brackets and included adults and children, only studies that could explicitly assess the impact on children and adolescents were taken into account.

Concerning the parameters of glycemic control, we considered studies that provided information on glycated hemoglobin (HbA1c) levels before and after the treatment. We also included those studies that conducted an OGTT and gave information about glucose, insulin, and C-peptide levels. If the oral glucose insulin sensitivity (OGIS) index was used in a certain study, we also included it. Additionally, when available, we considered continuous glucose monitoring (CGM) data, as well as eventual changes in insulin dosage after the treatment.

### Clinical trial

Based on Bellin et al. small pilot study, demonstrated that in a 14-year-old male patient with newly diagnosed CFRD, who had not received insulin treatment during the study or beforehand, daily oral ivacaftor therapy over 4 weeks did not enhance the acute, rapid-phase insulin response, measured by intravenous glucose tolerance test (IVGTT). The therapy significantly improved the prolonged insulin response during the 2-hour OGTT. At baseline, his OGTT insulin secretion (AUC) was 1,485 mU/L·min, which nearly doubled to 2,955 mU/L·min post-treatment. His glycemic category remained unchanged in terms of OGTT glucose levels, despite the improvement in insulin secretion. The authors suggested that improved prolonged insulin secretion despite a persistently blunted rapid-phase response may reflect a relatively preserved residual β-cell mass in the early stages of CFRD. Consequently, early adjustment of treatment with CFTRm in pediatric patients may delay or even prevent the progression of diabetes in cystic fibrosis by leveraging this preserved β-cell function (8). The study's limitations include a pediatric population consisting of only two individuals, with only one diagnosed with CFRD immediately before the study. This limited representation makes it challenging to generalize the findings to a broader pediatric CFRD population. Additionally, the 4-week follow-up period may not be sufficient to capture long-term outcomes or the sustainability of ivacaftor's effects on insulin secretion.

### Observational studies

A study conducted by Li et al. showed that introducing lumacaftor-ivacaftor (LUMA/IVA) therapy does not significantly improve glucose control in this population, contrasting with earlier studies involving ivacaftor in G551D mutation carriers, which showed better outcomes. However, HbA1c level and fasting glucose level did significantly increase compared to the stage before therapy. There was a slight improvement in the male group, which emphasised the need for further studies (9).

In the observational study conducted by Korten et al. there is shown the effect of elexacaftor-tezacaftor-ivacaftor (ETI) use on glucose tolerance in sixteen adolescents with pancreatic insufficiency due to CF. The median age of the participants was 13.8 years. The study aimed to assess the changes in glucose metabolism that occurred during CFTRm therapy. OGTT was performed before and 4–6 weeks after the start of therapy in 15 participants, which allowed the measurement of plasma glucose, serum insulin, C-peptide, and HbA1c levels. Before starting ETI therapy, 2 patients were diagnosed with CFRD, and after the end of treatment, none of the patients met the criteria for CFRD. Glucose metabolism improved in seven patients, remained stable in another seven, and worsened in one patient. Plasma glucose concentration after 60 min (mmol/L) before ETI therapy = 10.86 (9.61; 12.48), after = 9.74 (8.28; 11.62) for [*p* = 0.03]. Plasma glucose concentration after 90 min (mmol/L) before ETI therapy = 9.62 (7.63; 11.38) after = 9.08 (6.5; 10.18) for [*p* = 0.04]. Plasma glucose concentration after 120 min (mmol/L) before ETI therapy = 7.67 (5.9; 9.35) after = 5.78 (4.9; 7.2) for [*p* = 0.03] After the end of ETI therapy, insulin levels (mU/L) were lower at 120 min [before ETI = 58 (33.2; 79.5), after = 32.65 (14.1; 45.3) *p* = 0.01] and 180 min [before ETI = 13.4 (8.6; 24.1), after = 8.7 (4.9; 12.1) (*p* = 0.006)]. ETI therapy also had a positive effect on C-peptide levels at 180 min of the test (ng/ml) [before ETI = 3.52 (2.09; 4.97), after = 2.02 (1.58; 3.18) (*p* = 0.005)]. HbA1c did not change after the end of therapy. CGM) was implemented on 11 participants starting 3 days before treatment and continuing through day 7 of ETI treatment. Mean, minimum, and maximum glucose levels were calculated before and after initiation of the treatment, and no difference was found (10).

### Case reports

The case report presented by Park et al. investigates the impact of the ETI in the UK in August 2020 on children over 12 years old diagnosed with CFRD and possessing a CFTR gene mutation for which the treatment was approved. Glycemic control was monitored using the CGM system 5 days before treatment initiation and continued for 14 days. Key metrics included time in the target glucose range (3–10 mmol/L), percentage of time spent in hypoglycemia (<3 mmol/L), and hyperglycemia (>10 mmol/L), alongside insulin dosage requirements. Patients were monitored at least every three months, with CGM data collected every six months or sooner if necessary. Out of seven participants of treatment with ETI, four of them (57.1%) were able to discontinue insulin therapy entirely. Two additional patients were able to temporarily cease insulin administration, later resuming treatment after 2 and 10 months, respectively, with reduced dosage requirements (from 25 units/ day to 1 unit/day and from 2 units/day to 0.5 units/day). One individual required an escalation in both basal and bolus insulin doses. Glycemic control remained stable or improved despite the reduction or cessation of insulin, indicating enhanced endogenous glucose regulation. Some participants who discontinued insulin experienced episodes of hypoglycemia, underscoring the need for careful monitoring (1). The initiation of ETI therapy in children and adolescents with CFRD appears to positively influence glycemic control, potentially reducing or eliminating the need for exogenous insulin. However, the occurrence of hypoglycemia in some patients post-insulin discontinuation highlights the necessity for vigilant glucose monitoring and patient education regarding hypoglycemia recognition and management.

The case report presented by Stekolchik et al. described the case of a 16-year-old female with a G85E gene mutation, diagnosed at birth with meconium ileus, pancreatic insufficiency, and CFRD. The patient was treated with CFTRm, and the effect of ETI on clinical parameters was studied. For this purpose, they were measured before the start of therapy and 10 months after its initiation. The initial HbA1C level was 7.8%, and after the elapsed time, it was 8.7% (11). Considering the weight gain observed after CFTRm in this patient, there is a need for further studies to understand the metabolic effects of the therapy. However, it cannot be ruled out that the increase in the glycated hemoglobin level in this patient was influenced by the patient's nutritional status.

## Discussion

Although several studies have investigated CFTR potentiator therapy in cohorts that include pediatric patients, the available data are often derived from mixed-age populations. As a result, most outcomes are presented as median values for the entire cohort, limiting the ability to draw conclusions specific to children and adolescents. The available evidence, while generally indicating improvements in insulin secretion and glycemic control, must therefore be interpreted with caution due to heterogeneity in age distribution, study design, and outcome measures (12–15). In addition to the studies included in our review, some reported important insights into the topic.

The case report by Tsabari et al. investigated the effects of ivacaftor in young adults (under 25 years old) with CFRD and indeterminate glycemia (INDET), focusing on two sibling patients. The study compared glycemia and insulin levels before and after 16 weeks of ivacaftor therapy using an OGTT. It offers an important mechanistic insight by illustrating how ivacaftor may improve glycemic control and β-cell responsiveness, even over a short treatment period. The first patient, a 24-year-old male, showed improved blood glucose levels. His fasting blood sugar level decreased after therapy, with 1-hour levels changing from 209 mg/dl to 198 mg/dl and 2-hour levels dropping from 77 mg/dl to 43 mg/dl. His fasting insulin levels increased and levels of insulin at 30 min rose from 108 pmol/L to 140 pmol/L. However, 2-hour insulin levels were lower after therapy, dropping from 193 pmol/L to 90 pmol/L. The second patient is a 22-year-old female who was diagnosed with CFRD at 21. Her fasting blood sugar level decreased after treatment also the OGTT insulin measurement in 2 h dropped from 367pmol/L to 268 pmol/L. However, the 1-hour insulin level increased from 8 pmol/L to 129 pmol/L. Nevertheless, its single-case nature and short follow-up preclude broader generalization (16). In contrast, the multicenter study by Wood et al. included a relatively large sample (*n* = 175) and demonstrated statistically significant reductions in HbA1c after ETI therapy, accompanied by improvements in nutritional and pulmonary parameters. However, despite these metabolic benefits, the lack of correlation between HbA1c and lung function metrics, along with no significant alterations in CGM data glucose metrics, suggests that the metabolic response to ETI may be multifactorial and not solely driven by pulmonary improvements (17).

Bayona et al. further emphasized the importance of baseline pancreatic reserve, demonstrating that CFTR modulators may enhance glucose metabolism predominantly in patients with preserved β-cell function, while those with established CFRD exhibit limited benefit. The CFRD patients' baseline OGTT results showed higher glucose AUC values, impaired insulin secretion, and reduced fasting glucose at 52 weeks compared to other subgroups. No significant changes in insulin or C-peptide AUC were noticed. The CGM data over the 52 weeks showed that the CFRD subgroup, when compared to those with normoglycemia and hyperglycemia, continued to exhibit a higher percentage of time above range (TAR > 180 mg/dl). Notably, HOMA-IR increased (*p* = 0.003). Glycemic progression varied: 3 remained CFRD, 2 improved to IGT, and 2 IGT patients progressed to CFRD on the treatment. This highlights potential variability in the mechanism of action depending on the degree of pancreatic damage. The observed increase in HOMA-IR suggests a compensatory rise in insulin resistance, warranting further mechanistic investigation (13).

Small-scale studies, such as those by Thomassen et al. and Dagan et al., reinforce the notion that individual metabolic responses to CFTR modulators are highly variable. Differences in genotype (e.g., F508del homozygosity vs. S549R mutation), treatment duration, and age likely contribute to these discrepancies. For example Thomassen et al. showed that overall, no significant improvement in glucose metabolism or insulin secretion was observed after the treatment of 5 Phe508del-homozygous CF patients (ages 13–33) with LUMA/IVA for over 6–8 weeks. Dagan et al. also presented that after the treatment, HbA1c levels in the overall cohort did not significantly change (18).

Moreover, the small number of pediatric participants and short observation ranges limits the interpretative power of these studies (18, 19). Similarly, Bassi et al. observed transient improvements in total daily insulin dose (TDD) observed at 3 months (*p* = 0.01) and 6 months (*p* = 0.04), following ETI initiation, but these effects were not sustained at 12 months, indicating possible adaptation over time (12). Although pediatric patients were a part of this study, no separate statistical evaluation was performed for this group as its sample size was small.

Although the study by Misgualt et al. is limited by its non-randomized design, small sample size of only 9 young participants, and issues with drug intolerance, it provides valuable insights through detailed oral glucose tolerance test (OGTT) analysis. The findings indicate an early positive effect of CFTR modulators on the treatment of glucose abnormalities in youth (14). However, Moheet et al. found minimal effects of LUMA/IVA therapy on glucose metabolism only in patients with CF homozygous for the F508del CFTR mutation over 12 months, with no significant improvements in the majority of subjects, underscoring the heterogeneity across studies (15). Collectively, these findings indicate that treatment effects may depend on baseline metabolic status, genotype, and drug combination, and that randomized controlled studies with age-stratified analyses are needed.

From a mechanistic standpoint, CFTR correction might influence β-cell function and insulin secretion directly, or indirectly through improvements in systemic inflammation, nutrition, and pulmonary status. However, the variability in results across studies suggests that the relationship between CFTR function and glucose metabolism is complex and influenced by multiple confounding factors, including insulin resistance, disease stage, and treatment duration.

Although studies such as Piona et al. have demonstrated substantial pulmonary improvements with ETI, metabolic outcomes remain inconsistent. The decline in insulin sensitivity following LUMA/IVA and the stable glucose metabolism observed after ETI suggest that newer modulators do not consistently confer metabolic benefits. Insulin sensitivity, assessed using the OGIS index, declined significantly after LUMA/IVA treatment, with a median reduction of 15.8% [IQR −11.1% to −23.3] compared to baseline. In contrast, no significant change in OGIS was observed in participants receiving ETI. Insulin availability was marginally lower than expected at both time points, but these differences were not statistically significant (20). This emphasizes the need for prospective pediatric trials employing sensitive methods (CGM, IVGTT) to better delineate these effects.

The main strength of the presented review is that, to the best of our knowledge, this is the first such summary attempting to draw conclusions from studies investigating the impact of CFTRm therapy on carbohydrate metabolism disorders solely in the pediatric population. In one of recently published systematic reviews by Giordiano et al. authors analyzed an impact of CFTRm on glucose metabolism in patients with CFRD but in a more diverse group, including young adults (21). The innovative aspect of focusing exclusively on pediatric and adolescent populations is the inclusion of patients mainly in the early phases of CFRD. In this population, CFTRm therapy may exert the greatest potential benefits in the field of beta-cell function and insulin regulation.

The main limitation of presented review is the small number of included studies, as well as the sizes of their samples. Another shortcoming can be the diversity in outcomes the reviewed studies assessed.

## Conclusions

In summary, current evidence indicates that CFTR modulators hold potential to positively affect glucose metabolism in cystic fibrosis, particularly when introduced before significant pancreatic β-cell loss occurs. Additionally, it's important to determine the optimal type of drug and the best timing for intervention, taking into account the metabolic status of age-specific patients with CFRD. Most of the data reported above were trends rather than statistically isolated findings, due to their limitations. The data presented in this systematic review, along with findings from studies involving mixed-age groups, highlight the need for larger-scale and long-term investigations, especially clinical trials, focused exclusively on pediatric populations. Such studies are crucial to verify the long-term effectiveness and impact of CFTRm therapy on CFRD.
